# From pressure in the pipeline to accelerating ascension: a survey to understand professional experiences of and opportunities for Canadian women in the healthcare sector

**DOI:** 10.1186/s12960-023-00800-0

**Published:** 2023-02-20

**Authors:** L. Desveaux, J. Pirmohamed, N. Hussain-Shamsy, C. Steele Gray

**Affiliations:** 1 Institute for Better Health, Trillium Health Partners, 100 Queensway West, Mississauga, ON Canada; 2 Institute for Health Policy, Management, and Evaluation, University of Toronto, 155 College St, Toronto, ON Canada; 3 Child Health Evaluative Sciences, Sick Kids Research Institute, 666 Bay St, Toronto, ON Canada; 4 Lunenfeld-Tanenbaum Research Institute, Sinai Health, 14 St. Matthews Road, Toronto, ON Canada

**Keywords:** Gender equity, Survey, Policies, Programs, Healthcare, Women

## Abstract

**Background:**

Much has been written about the state and persistent lack of progress regarding gender equity and the commonly referenced phenomenon of a ‘leaking pipeline’. This framing focuses attention on the symptom of women leaving the workforce, rather than the well-documented contributing factors of hindered recognition, advancement, and financial opportunities. While attention shifts to identifying strategies and practices to address gender inequities, there is limited insight into the professional experiences of Canadian women, specifically in the female-dominated healthcare sector.

**Methods:**

We conducted a survey of 420 women working across a range of roles within healthcare. Frequencies and descriptive statistics were calculated for each measure as appropriate. For each respondent, two composite Unconscious Bias (UCB) scores were created using a meaningful grouping approach.

**Results:**

Our survey results highlight three key areas of focus to move from knowledge to action, including (1) identifying the resources, structural factors, and professional network elements that will enable a collective shift towards gender equity; (2) providing women with access to formal and informal opportunities to develop the strategic relational skills required for advancement; and (3) restructuring social environments to be more inclusive. Specifically, women identified that self-advocacy, confidence building, and negotiation skills were most important to support development and leadership advancement.

**Conclusions:**

These insights provide systems and organizations with practical actions they can take to support women in the health workforce amid a time of considerable workforce pressure.

**Supplementary Information:**

The online version contains supplementary material available at 10.1186/s12960-023-00800-0.

## Background

It is well established that women are consistently underrepresented in governance and leadership positions in healthcare [1], despite being equally driven early in their careers. This underrepresentation is due to patterns of hindered recognition (e.g., awards and speaking engagements) [2–5], advancement (e.g., promotion) [6], and financial opportunities (e.g., compensation) [7, 8]. These drivers contribute to the commonly referenced phenomenon of a ‘leaking pipeline’ [9], which focuses attention on the symptom of women leaving the workforce. However, this detracts the focus from the root cause: the inter-related factors that contribute to highly skilled women stagnating in their progress or leaving their careers. Indeed, this phenomenon is driven by the interplay between the above-referenced structural factors (unequal division of power between women and men) [10], available resources, and unconscious bias that maintains them [2–5, 7, 8], which in turn have an impact on the individual themselves.

The business case for gender equity is compelling—when organizations have greater representation of women, they perform better financially [11, 12] and women leaders tend to better respond to and invest in the communities they serve [13, 14]. Encouragingly, attention has shifted to the need to identify organizational strategies and structural practices that will address gender inequities that lead to the ‘leaking pipeline’ [15–17]—ideally a multilevel organizational approach that targets organizational processes, awareness and engagement, mentoring and networking, and leadership development and support tools [18]. In parallel, women have highlighted the need to acknowledge common challenges and validate their experiences [19]. Most of the healthcare literature attending to gender equity in managerial roles to date has emerged from the United States. Several Canadian studies have highlighted the extent of gender inequity [6, 20–22]; however, none have sought to broadly characterize the experience of Canadian women.

Further, most work in the healthcare space has focused on physicians [23], who represent a fraction of the global health workforce [24] and only 10% of the Canadian health professional workforce [25]. Healthcare is provided by a long list of professionals, including nurses, midwives, pharmacists, allied health professionals, social workers, and paramedics, among others, and is broadly supported by a range of non-clinical roles, including researchers, administration, and enabling functions (e.g., marketing, finance, human resources, etc.).

To address these gaps, we undertook a web-based survey to understand the specific experiences of Canadian women working across the healthcare sector and their perspectives on the types of supports needed to address existing gaps. Insights from this study can support the focused co-design of organizational and system-level strategies to target the specific experiences and preferences of women in healthcare.

## Methods

We conducted an open, cross-sectional electronic survey from January 25 to April 8, 2021. This manuscript follows the reporting requirements outlined in the Checklist for Reporting Results of Internet E-Surveys (CHERRIES) [26]. This initiative was formally reviewed by institutional authorities at Women’s College Hospital and was deemed not to require Research Ethics Board approval (File # 2020-0125-E).

### Recruitment and data collection

The survey asked respondents ‘What gender do you identify with?’, with available responses including male, female, other (please specify), and prefer not to disclose (see Additional file 1). Individuals who self-identified as a woman were eligible to participate in the e-survey. A convenience sampling approach to recruitment was utilized whereby details, including a URL link to complete the survey, were distributed through several mechanisms, including posts on social media (Twitter and LinkedIn accounts), the mailing list of Women Who Lead (a volunteer Women’s Leadership Organization (www.women-who-lead.com), and targeted emails to key gender equity organizations, and health leadership organizations. The research team also connected to health system leaders who were encouraged to share details of the e-survey with their networks.

After self-referral to the study, informed consent was obtained digitally from eligible participants before accessing the e-survey. Participants could enter their name into a draw to win a 1-h session with a career coach as a recruitment incentive and could provide their email address to be contacted for future research, but otherwise no identifying information was collected. Contact information was stored separately from study data.

The e-survey was administered through Qualtrics, a secure Web platform for building and managing online surveys. Data were only accessible to authorized individuals on the study team. The e-survey took 15–20 min to complete and incorporated adaptive questioning to reduce the number and complexity of questions. All survey questions were optional. Usability, comprehensiveness, and technical functionality were piloted with four women in healthcare before launching the e-survey.

### Instrument design and measures

#### Individual and structural factors

Individual factors are those specific to the individual themselves and can impact career development, including factors related to human capital, career experiences, career self-management, and motivation [27–30]. Items that capture these elements were drawn from two career development surveys—a validated questionnaire assessing career competencies [31–33] and a widely distributed survey on women’s leadership [31–33]. All factors were rated on a 5-point Likert scale (i.e., strongly agree to strongly disagree). Participants were also provided with a list of training and development resources and asked to indicate (i.e., select all that apply) those which they believed will help women move into leadership roles in the future [31].

Structural factors are those related to processes, policies, cultural norms, and values at the organizational level that can impact career patterns by creating an enabling or disabling environment for career advancement [27]. Questions were adapted from the several questions from the Conditions for Work Effectiveness Questionnaire [34] with content informed by the literature on structural factors that impact career progression [27, 35]. Specifically, questions were modified to ask participants to rate access to these structural factors within their workplace (e.g., opportunities for feedback, financial benefits, career development resources, advancement opportunities, etc.).

#### Unconscious bias

Unconscious bias refers to “the attitudes or stereotypes that affect our understanding, actions, and decisions in an unconscious manner, which are activated involuntarily, without awareness or intentional control” [36] and is often activated by situational cues [37, 38]. We assessed the perceived experience of unconscious biases by asking participants to indicate on a 5-point Likert scale (i.e., strongly agree to strongly disagree) whether they perceived promotions in their organization were based on fair and objective criteria. In addition, we adapted questions from Lean In Canada’s 2019 Women in the Workplace Survey [39] to assess whether participants perceived equal access to career development, advancement, and sponsorship opportunities compared to their male colleagues, as well as whether they needed to provide more evidence of their competence or had their judgement questioned more often.

#### Available resources

This series of questions assessed access to support, resources, mentors, or sponsors to assist with career development and advancement [39]. Participants were asked to indicate on a 5-point Likert scale (i.e., strongly agree to strongly disagree) whether they had access to mentorship (i.e., someone who provides advice, feedback, and coaching to foster development) [40] and sponsorship (i.e., someone who through advocates, protects, and fights for career advancement) [41] as well as whether they had access to someone who helps them manage their career path, navigate organizational politics, and advocate for new opportunities for them. In addition, participants were asked to indicate whether their manager gives them opportunities to manage projects, manage colleagues, to showcase their work to colleagues who are comparable and senior to them in terms of their role and skills [39]. Participants were also given the option to indicate “Not Applicable”.

### Analysis

Frequencies and descriptive statistics were calculated for each measure as appropriate. For each respondent, two composite Unconscious Bias (UCB) scores were created using a meaningful grouping approach [42] based on responses to seven questions related to perceptions of bias in the workplace—one score using five responses of questions of bias compared to male colleagues (i.e., responses to the question, “compared to my male colleagues who are comparable to me in terms of role and skills…”) and the other to female colleagues (i.e., responses to the question, “compared to my female colleagues who are comparable to me in terms of role and skills…”), and an additional two questions on bias in the workplace in general. Individual scores for each question were reported on a 5-point Likert Scale (i.e., strongly agree to strongly disagree), reversed-coded if appropriate, and summed for a total possible score of 35, with a higher score indicating a greater perception of bias. Each of the seven components of bias were weighted equally as there was no conceptual reason to weight any one component high than another. UCB scores for male (UCB-Male) and female (UCB-Female) colleagues were compared to each other, and also each compared to access to structural resources in the respondent’s present job/organization using a paired samples t test. Individual factors related to career growth and development and its correlation to UCB score for female and male colleagues were conducted using a Spearman’s coefficient. Significance for all tests was set to *p* < 0.05.

## Results

Of the 517 unique individuals (i.e., unique IP address) accessed the survey, 31 were ineligible (did not consent to the survey or did not self-identify as a woman), resulting in a final sample of 486 participants. The survey completion rate (defined as the number who finished the last page of the survey divided by the number who agreed to participate) was 73.8%. Not all 486 respondents completed all survey questions, leading to different response counts across questions due to missing data. Data from all questionnaires were included in analysis regardless of survey completion status.

Participants were mostly between 25 and 44 years old (*n* = 356, 73.3%), and represented a diverse range of ethnic backgrounds (Table 1). Our sample skewed Canadian (*n* = 447, 96.7%; two-thirds were Canadian born, *n* = 290, 65.9%); most of whom lived in Ontario (*n* = 408, 91.5%). The majority were married or in a common-law relationship (*n* = 328, 67.5%), and just less than half had a child(ren) or dependent(s) (*n* = 225, 46.3%). Only 37 (7.6%) identified as having a disability. Refer to Table 1 for full details.

**Table 1:** Sociodemographic profile

	*N*^a^ (%)
Age	
18–24 years	12 (2.5)
25–34 years	168 (34.6)
35–44 years	188 (38.7)
45–54 years	89 (18.3)
55 + years	29 (6.0)
Ethnic or cultural group^b,c^	
North American Aboriginal	*n.r*
Other North American	132 (27.2)
European	210 (43.2)
Caribbean	12 (2.5)
Latin, Central, South American	15 (3.1)
African	10 (2.1)
Asian	109 (22.4)
Oceanian	*n.r*
Not Sure	8 (1.6)
Prefer not to disclose	11 (2.3)
Other	11 (2.3)
Place of residence	**467**
Maritimes, Canada	6 (1.2)
Quebec, Canada	12 (2.7)
Ontario, Canada	408 (91.5)
Manitoba, Canada	5 (1.1)
Alberta, Canada	8 (1.8)
British Columbia, Canada	7 (1.6)
United States	15 (3.2)
Other	5 (1.1)
Immigration status	**440**
Canadian-born	290 (65.9)
Immigrant	150 (34.0)
< 5 years in Canada	6 (1.4)
5–10 years in Canada	12 (2.7)
> 10 years in Canada	132 (30.0)
Marital status	**462**
Single	77 (15.8)
In a relationship	28 (5.8)
Common law or married	328 (67.5)
Divorced, separated, widowed, or other	29 (6.0)
Has children or dependents	**463**
Yes	**225 (46.3)**
Has a disability	**324**
Yes	**37 (7.6)**

^a^*n* = 486, unless otherwise specified

^b^“Select all that apply” response format

^c^Note: small cell sizes (*n* < 5) for ethnic group are not reported (*n.r.*)

^d^Maritime provinces defined as: Newfoundland and Labrador, Nova Scotia, New Brunswick, and PEI

Professionally, most had either a non-clinical, graduate-level degree (e.g., MSc, MHA, MBA, etc.; *n* = 189, 38.9%) or an undergraduate degree or less (*n* = 136, 28.0%). Career stages were well represented by our sample, with the majority being early career (*n* = 210, 43.2%). Most respondents worked in a hospital environment (*n* = 314, 64.6%), academic institution (*n* = 59, 12.1%), or public agency (e.g., government or community agency, *n* = 43, 8.8%). Of the 369 participants who responded to the question, the majority aspired to be in a leadership position (*n* = 201, 54.5%), while approximately one in five were either unsure (*n* = 44, 11.9%) or did not aspire to a leadership position (*n* = 39, 10.6%). Refer to Additional file 1: Table S1 for further details.

### Workplace experiences

*Unconscious bias*: A UCB score was calculated for 422 respondents for whom enough data were available. Respondents perceived significantly more unconscious bias when comparing themselves to male colleagues in a similar role than when comparing female colleagues in a similar role [mean (standard deviation, SD) scores: UCB-male: 22.3 (5.7); UCB-female: 19.9 (5.2), *p* < 0.001]. Refer to Table 2 for complete results.

**Table 2:** Perceptions of unconscious bias in the workplace

	Compared to male colleagues with a similar role and skills	Compared to female colleagues with a similar role and skills	
I have equal opportunity for growth and development (*n* = 436)			< 0.001*
Strongly agree	42 (8.6)	75 (15.4)	
Agree	120 (24.7)	221 (45.5)	
Neutral	100 (20.6)	70 (14.4)	
Disagree	125 (25.7)	60 (12.3)	
Strongly disagree	50 (10.3)	13 (2.7)	
I have equal opportunity for advancement in my organization (*n* = 433)			< 0.001*
Strongly agree	40 (8.2)	59 (12.1)	
Agree	104 (21.4)	189 (38.9)	
Neutral	116 (23.9)	92 (18.9)	
Disagree	131 (27.0)	79 (16.3)	
Strongly disagree	45 (9.3)	17 (3.5)	
I have equal access to sponsorship in my organization (*n* = 433)			< 0.001*
Strongly agree	35 (7.2)	54 (11.1)	
Agree	90 (18.5)	134 (27.6)	
Neutral	156 (32.1)	153 (31.5)	
Disagree	108 (22.2)	71 (14.6)	
Strongly disagree	45 (9.3)	24 (4.9)	
I have needed to provide more evidence of my competence (*n* = 435)			< 0.001*
Strongly agree	90 (18.5)	52 (10.7)	
Agree	152 (31.3)	116 (23.9)	
Neutral	94 (19.3)	110 (22.6)	
Disagree	71 (14.6)	127 (26.1)	
Strongly disagree	28 (5.8)	33 (6.8)	
I have had my judgement questioned in my area of expertise. (*n* = 436)			< 0.001*
Strongly agree	87 (17.9)	44 (9.1)	
Agree	147 (30.2)	142 (29.2)	
Neutral	84 (17.3)	79 (16.3)	
Disagree	89 (18.3)	136 (28.0)	
Strongly disagree	30 (6.2)	38 (7.8)	
Promotions are based on fair and objective criteria (*n* = 436)			
Strongly agree	11 (2.5)		
Agree	96 (22.0)		
Neutral	188 (43.1)		
Disagree	105 (24.1)		
Strongly disagree	36 (8.3)		
I can see my trajectory forward in my organization (*n* = 459)			
Strongly agree	29 (6.3)		
Agree	131 (28.5)		
Neutral	150 (32.7)		
Disagree	115 (25.1)		
Strongly disagree	34 (7.4)		
Unconscious Bias Score^a^ (*n* = 422) mean (SD)	22.3 (5.7)	19.9 (5.2)	< 0.001*

Self-described perceptions of bias, compared between male and female colleagues, comparable in terms of role and skill, as *n* (%) unless otherwise specified

^a^A composite unconscious bias score was created where responses for each unconscious bias question were summed together (strongly agree = 1, strongly disagree = 5 with reverse coding as appropriate). Based on a total possible score of 35, a higher score indicates greater perception of unconscious bias. Groups were compared using a paired samples *t* test

*Available resources*: Respondents perceptions of availability of and access to career development resource in the workplace varied across the sample. Approximately 40–50% of the participants did not have access to someone to help manage their career path (disagree or strongly disagree: *n* = 202, 49.4%), advocate for new opportunities (disagree or strongly disagree: *n* = 183, 44.4%), or navigate organizational politics (disagree or strongly disagree: *n* = 193, 39.7%). Most noted that they were given opportunities to manage projects (agree or strongly agree: *n* = 234, 60.0%). More noted that their manager gave them opportunities to showcase their work to colleagues of comparable role and skill (agree or strongly agree: *n* = 198, 50.6%) than to those who are senior to them (agree or strongly agree: *n* = 168, 43.2%). Access to mentorship was slightly more frequently experienced (agree or strongly agree: *n* = 131, 32.6%; disagree or strongly disagree: *n* = 194, 48.3%) than access to sponsorship (agree or strongly agree: *n* = 83, 20.6%; disagree or strongly disagree: *n* = 194, 48.3%) for career advancement. Refer to Additional file 1: Table S2 for full results.

### Structural and individual factors

*Structural factors* (Table 3): Perceived access to financial benefits (e.g., medical benefits, tuition support; *n* = 288, 59.3%), and receiving specific feedback on strengths (*n* = 205, 42.2%), and areas for improvement (*n* = 205, 42.2%) were the most frequently reported structural resources available within one’s organization. Rewards and recognition for a job well done (*n* = 109, 22.4%), opportunity for advancement to a senior-level role (*n* = 105, 21.6%), and opportunities to network with executives external to the organization (*n* = 92, 18.9%) were perceived the least frequently available resources. Whether or not each structural factor was perceived as available to participants was significantly associated with both the respondent’s UCB-male and UCB-female score (p < 0.001).

**Table 3:** Self-described access to structural factors influencing career progression

	*N* (%)
Access to financial benefits (e.g., medical benefits, tuition support)	288 (59.3)
Specific feedback about your strengths	205 (42.2)
Specific feedback about things you could improve on	205 (42.2)
Skills building outside your area of expertise/training	173 (35.6)
Access to job training and development resources	164 (33.7)
Opportunities to network with executives within the organization	155 (31.9)
Access to professional career development resources	148 (30.5)
Access to non-financial benefits (e.g., child care, flexible schedule)	121 (24.9)
Rewards and recognition for a job well done	109 (22.4)
The opportunity to advance to a more senior-level job	105 (21.6)
Opportunities to network with executives external to the organization	92 (18.9)

*Individual factors* (Table 4): One-third of respondents noted that they did not see their themselves reflected by senior leaders in their organization (disagree or strongly disagree: *n* = 145, 33.8%). However, the majority noted that they were able to be their authentic self at work (agree or strongly agree: *n* = 264, 61.4%). Responses to both these questions were highly correlated with the UCB-male and UCB-female score. Most respondents identified that they know what is important to them in their career (*n* = 366, 86.5%).

**Table 4:** Individual factors

	*N* (%)	UCB	
		Women colleagues	Men colleagues
I see myself reflected by senior leaders in my organization (*n* = 429)		0.41 (416)*	0.42 (415)*
Agree or strongly agree	182 (42.4)		
Neither agree nor disagree	102 (23.8)		
Disagree or strongly disagree	145 (33.8)		
I usually search for detailed information about the professional area and jobs in which I am interested (*n* = 428)		− 0.03 (415)	0.07 (414)
Agree or strongly agree	320 (65.8)		
Neither agree nor disagree	54 (11.1)		
Disagree or strongly disagree	54 (11.1)		
I am very clear about my career development goals (*n* = 428)		0.4 (415)	0.03 (414)
Agree or strongly agree	216 (50.5)		
Neither agree nor disagree	114 (26.6)		
Disagree or strongly disagree	98 (22.9)		
I usually seek out opportunities for helpful career guidance (*n* = 427)		0.3 (414)	0.03 (413)
Agree or strongly agree	271 (63.5)		
Neither agree nor disagree	79 (18.5)		
Disagree or strongly disagree	77 (18.0)		
I know what is important to me in my career (*n* = 423)		− 0.03 (411)	− 0.05 (409)
Agree or strongly agree	366 (86.5)		
Neither agree nor disagree	44 (10.4)		
Disagree or strongly disagree	13 (3.1)		
I am able to be my authentic self at work (*n* = 430)		0.4 (416)*	0.39 (415)*
Agree or strongly agree	264 (61.4)		
Neither agree nor disagree	73 (17.0)		
Disagree or strongly disagree	93 (21.6)		

Question included a “select all that apply” response format

*p < 0.001

*Leadership skills* (Table 5): Respondents identified that self-advocacy, confidence building, and negotiation skills were most important for helping women move into leadership roles in the future (*n* = 309, 63.6%; *n* = 304, 62.6%; and *n* = 274, 56.4% respectively).

**Table 5:** Skills that will support women moving into leadership roles

	*N* (%)
Self-advocacy	309 (63.6)
Confidence building	304 (62.6)
Negotiating	274 (56.4)
Networking	253 (52.1)
Articulating and sharing a point of view	210 (43.2)
People management	206 (42.4)
Decision-making	191 (39.3)
Financial/budgeting for my professional role	175 (36.0)
Team building	145 (29.8)
Project management	136 (28.0)
Talent management	125 (25.7)
Social interaction/collaboration	116 (23.9)
Critical thinking	116 (23.9)

## Discussion

The survey highlights significant perceptions of unconscious bias among over 400 women working in the health sector across a range of roles. Perceptions of bias were significantly associated with perceived fit in the organization—specifically the ability to see themselves reflected in leadership and the ability to be authentic at work. Despite healthcare being a female-dominated industry, women experience a significant degree of unconscious bias compared to their male colleagues. Most women indicated having a strong sense of what was important to them and actively seeking out opportunities for career guidance, yet they lacked access to conversations about career development and opportunities for growth within their organization. Participants identified a need to build relational skills to support their advancement and leadership development, including self-advocacy, confidence building, negotiating, networking, and articulating their point of view. It is unclear from our data whether opportunities to develop these skills do not exist within their organizations, whether awareness or access is generally limited, or whether women experience challenges navigating available resources.

Perceived access to resources was highly variable with 23–49% of respondents reporting lack of access, underscoring the importance of identifying the resources and structural factors that will enable a collective shift towards gender equity. Specifically, there is a need to objectively identify what resources women have access to and how they are supported in the workplace. Women routinely reported having their judgement questioned, being required to provide evidence of their competence, and having fewer opportunities for advancement compared to their male colleagues [43]. As a societal system of cultural beliefs, gender shapes occupational contexts, what individuals believe about themselves and others, and how individuals interact with one another [44]. These cultural beliefs can impede professional development not only by requiring women to work harder to advance, but also by making it more difficult for women to figure out how to be an effective leader in way that is compatible with their gender [45] in contexts where there is a relative lack of women role models who might support them in navigating this process [46]. The fact that respondents in our survey noted a lack of role models in their organizations suggests that they may likely be experiencing this difficulty as well.

The top five skill women identified to support advancement and leadership development centre around communication, highlighting the need to attend to professional networks and ensure that women have access to formal and informal opportunities to develop the strategic relational skills required for advancement. That self-advocacy and confidence topped the list helps contextualize the observation that women in healthcare are less likely to seek out elected positions due to a lack of experience and discomfort with self-promotion [47]. These collective insights represent a step towards identifying the resources, structural factors, and professional support that will enable a collective shift towards gender equity. Social environments within organizations can be restructured to support gender-equitable participation and advancement by the implementation of policies and practices that explicitly engage women to address the widespread experiences of unconscious bias and persistent perception of unequal access to resources identified in this study and numerous others [2–5, 7, 8]. The need for equal access to resources requires ongoing attention to and evaluation of: (1) *how* organizations and departments distribute human and material resources, including the informal practices that contribute to professional development; and (2) *what* specific structures are in place to ensure gender equity and *whether* they are effective in addressing gender inequities. For example, organizations can create structures that facilitate open communication and exchange among team members as well as creating opportunities for more junior staff to integrate with the existing network in environments that are highly collaborative. Organizations can also utilize gender impact assessments, gender-specific targets, gender tools of analysis, and identify gender-sensitive indicators as a mechanism to monitor progress [48]. While organizational leadership commitment and accountability is critical, implementing a comprehensive range of evidence-based strategies (e.g., flexible meeting policies, a corporate code of conduct, targets and quotas, programs to broaden experience, etc.) is necessary for change [18]. As policies are implemented, monitoring progress and impact is essential to avoid the presumption of fairness simply due to the introduction of revised policies [49].

Finally, our findings around unconscious bias support the well-established need to restructure social environments to be more inclusive. Compared to women, men have likely navigated through a greater breadth and size of informal networks through which they have learned shared norms around relational skills through their exposure to tacit knowledge [50]. Given that gender distributions are skewed at higher levels of leadership, men and women have differential opportunities to connect with same-gender colleagues in positions of relative influence and differential ability to utilize these contacts once developed [50]. This supports the hypothesis that while women have learned how to do their jobs effectively through training, they do not have equal access to the informal playbook on how to thrive in male-dominated networks and environments [51]. Creating structures and professional networks where women can come together—such as group coaching, a women’s leadership program, or a peer group—creates the mechanism for women to amplify one another, connect one another to opportunities, and compare notes, identify common experiences, and support one another’s learning [52, 53]. Approximately two-thirds of respondents reported a lack of mentorship in the current study—aligning with a well-document trend of limited support and mentorship in the literature [47, 54–56]—underscoring that prioritizing space and opportunities for mentorship is likely to have a positive impact on personal development, career choice, and productivity, and advancement opportunities [57].

### Limitations

Our study sample is heavily skewed towards those from Canada in general and Ontario specifically and may not reflect the experience of those in other regions. We also do not have information on rurality of residence, place of employment, or institutional size, which may additionally limit access to certain resources. While employing a convenience sampling recruitment strategy may limit broad reach, the study sample has diverse representation of age and career stage, as well as inclusion of those who work in healthcare in non-clinical roles. Further, while we used an established methodological approach to create the UCB composite score, it is a novel approach that has not been externally validated. Finally, this data focuses on participants’ perceptions of available opportunities and resources, which may not be an accurate reflection of resource availability. Future work should focus understanding the types of workplace structures and interactions that lead to participant experiences, objectively understanding the links between available resources, awareness, and access, and co-designing evidence-based strategies with women to ensure that they address the identified barriers.

## Conclusion

Reducing gender inequity is a persistent challenge facing the healthcare sector that has been exacerbated in recent years. Scholars—predominantly women—have made significant advancements in characterizing and describing current circumstances, yet relatively little progress has been made in identifying key system-level opportunities to promote the development and retention of women in healthcare more broadly. Our survey results highlight three key areas of focus to move from knowledge to action, including (1) identifying the resources, structural factors, and professional network elements that will enable a collective shift towards gender equity; (2) providing women with access to formal and informal opportunities to develop the strategic relational skills required for advancement; and (3) restructuring social environments to be more inclusive. We are hopeful that these insights provide systems and organizations with practical actions they can take to support women in the health workforce. Future scholarly work should focus on co-designing these strategies with women across career stages and evaluating their impact on confidence, networks, and advancement over time.

## Supplementary Information

**Additional file 1.** Supplementary data tables.

## Data Availability

The dataset analyzed during the current study is available from the corresponding author on reasonable request.
