# Supplementary material for: From pressure in the pipeline to accelerating ascension: a survey to understand professional experiences of and opportunities for Canadian women in the healthcare sector
Source: Hum Resour Health. 2023 Feb 20;21:12. doi: 10.1186/s12960-023-00800-0 (PMC9942417; doi:10.1186/s12960-023-00800-0)
Supplement: Supplementary file 1 — Additional file 1. Supplementary data tables. [file 12960_2023_800_MOESM1_ESM.docx]

**Supplementary Tables**

**Supplementary Table 1**: **Professional and Employment Information**

|  | | **N**^a^ **(%)** |
| --- | --- | --- |
| **Education^a^**  (n=484) | < Undergraduate degree | 136 (28.0) |
|  | Clinical Masters-level | 39 (8.0) |
|  | Non-clinical graduate-level | 189 (38.9) |
|  | PhD | 83 (17.1) |
|  | MD | 37 (7.6) |
| **Employment Status^b,c^** | Student | 36 (7.4) |
|  | Employed (Full-time) | 380 (78.2) |
|  | Employed (Part-time) | 36 (7.4) |
|  | Employed (Contract) | 44 (9.1) |
|  | Self-employed | 15 (3.1) |
|  | Other | 13 (2.7) |
| **Salary Range**  (n=467) | Less than $49,999 | 48 (9.9) |
|  | $50,000-$74,999 | 105 (21.6) |
|  | $75,000-$99,999 | 119 (24.5) |
|  | $100,000-$124,999 | 96 (19.8) |
|  | $125,000 or more | 83 (17.1) |
|  | Prefer not to disclose | 16 (3.3) |
| **Total Household Income**  (n=466) | Less than $49,999 | 14 (2.8) |
|  | $50,000-$74,999 | 45 (9.3) |
|  | $75,000-$99,999 | 45 (9.3) |
|  | $100,000-$124,999 | 60 (12.3) |
|  | $125,000 or more | 270 (55.6) |
|  | Prefer not to disclose | 32 (6.6) |
| **Career Stage**  (n=467) | Early Career | 210 (43.2) |
|  | Mid-level Career | 189 (38.9) |
|  | Senior Career | 68 (14.0) |
| **Employer**  (n=467) | Academic Institution | 59 (12.1) |
|  | Private Industry | 7 (1.4) |
|  | Public Agency^d^ | 43 (8.8) |
|  | Hospital | 314 (64.6) |
|  | Non-Hospital Healthcare Delivery Organization | 22 (4.5) |
|  | Self-employed | 8 (1.6) |
|  | Other | 14 (2.9) |
| **Nature of Work^e^**  (n=467) | Healthcare Provider | 139 (29.8) |
|  | Clinical Operations | 27 (5.6) |
|  | Clinical Management | 25 (5.4) |
|  | Research | 122 (26.1) |
|  | Administration | 84 (18.0) |
|  | Enabling Supports | 35 (7.5) |
|  | Clinician-Scientist or Clinician-Educator | 8 (1.7) |
|  | Other | 27 (5.8) |
| **Current or past leadership role^b^** | C-suite | 13 (2.6) |
|  | Vice President | 18 (3.7) |
|  | Director | 80 (16.5) |
|  | Senior Manager | 35 (7.2) |
|  | Other (i.e., Team, Project, or Departmental Lead) | 30 (6.2) |

^a^ < Undergraduate degree defined as: High school, college/trade school, or undergraduate degree; Clinical Masters-level education defined as: MScOT, MSW, or MScN; Non-Clinical graduate-level education defined as: Msc, MHA, MHSC, MBA, JD.

^b^ “Select all that apply” response format

^c^ “Other” defined as homemaker, on leave, on disability, retired, or other.

^d^ “Public Agency” defined as Government, Community Agency or Crown Agency

^e^ “Healthcare Provider” includes doctors, nurses and allied health providers; “Enabling supports” defined as Legal, Marketing, Communications, Accounting, Finance.

**Supplementary Table 2: Available Resources**

*Self-described access to resources, presented as N (%).*

|  | **Strongly Agree or Agree** | **Neither Agree nor Disagree** | **Disagree or Strongly Disagree** |
| --- | --- | --- | --- |
| I have access to someone to help manage career path. (n=409) | 154 (37.7) | 53 (13.0) | 202 (49.4) |
| I have access to someone who helps me navigate organizational politics. (n=410) | 158 (38.5) | 59 (14.4) | 193 (39.7) |
| I have access to someone who advocates for new opportunities for me. (n=412) | 167 (40.5) | 62 (15.0) | 183 (44.4) |
| My manager gives me opportunities to manage projects. (n=390) | 234 (60.0) | 66 (16.9) | 90 (23.1) |
| My manager gives me opportunities to manage my colleagues. (n=377) | 154 (40.8) | 60 (15.9) | 163 (43.2) |
| My manager provides opportunities for me to showcase my work to colleagues who are comparable to me, in terms of my role and skills. (n=391) | 198 (50.6) | 76 (19.4) | 117 (29.9) |
| My manager gives me opportunities to showcase my work to colleagues who are senior to me, in terms of my role and skills. (n=389) | 168 (43.2) | 82 (21.1) | 139 (35.7) |
| I have access to mentorship to advance my career. (n=402) | 131 (32.6) | 77 (19.2) | 194 (48.3) |
| I have access to sponsorship to advance my career. (n=402) | 83 (20.6) | 92 (22.9) | 194 (48.3) |
